# Hybrid Perovskites: Prospects for Concentrator Solar Cells

**DOI:** 10.1002/advs.201700792

**Published:** 2018-02-01

**Authors:** Qianqian Lin, Zhiping Wang, Henry J. Snaith, Michael B. Johnston, Laura M. Herz

**Affiliations:** ^1^ Department of Physics University of Oxford Clarendon Laboratory Parks Rd Oxford OX1 3PU UK; ^2^ School of Physics and Technology Wuhan University Wuhan 430072 P. R. China

**Keywords:** charge‐carrier recombination rates, concentrator solar cells, high solar irradiance, hybrid organic–inorganic perovskites, perovskite photovoltaics

## Abstract

Perovskite solar cells have shown a meteoric rise of power conversion efficiency and a steady pace of improvements in their stability of operation. Such rapid progress has triggered research into approaches that can boost efficiencies beyond the Shockley–Queisser limit stipulated for a single‐junction cell under normal solar illumination conditions. The tandem solar cell architecture is one concept here that has recently been successfully implemented. However, the approach of solar concentration has not been sufficiently explored so far for perovskite photovoltaics, despite its frequent use in the area of inorganic semiconductor solar cells. Here, the prospects of hybrid perovskites are assessed for use in concentrator solar cells. Solar cell performance parameters are theoretically predicted as a function of solar concentration levels, based on representative assumptions of charge‐carrier recombination and extraction rates in the device. It is demonstrated that perovskite solar cells can fundamentally exhibit appreciably higher energy‐conversion efficiencies under solar concentration, where they are able to exceed the Shockley–Queisser limit and exhibit strongly elevated open‐circuit voltages. It is therefore concluded that sufficient material and device stability under increased illumination levels will be the only significant challenge to perovskite concentrator solar cell applications.

## 1 Introduction

Perovskite solar cells have emerged over the last few years as highly promising photovoltaic applications, with power conversion efficiencies (PCEs) improving at an unprecedented rate. Recent optimized PCE values^1^ near 22.1% are now comparable with the most efficient thin film analogues, such as cadmium telluride and copper indium gallium selenide (CIGS) cells. Meanwhile, fundamental optoelectronic properties of hybrid perovskites have also been extensively investigated, revealing high charge‐carrier mobility and low recombination rates—key advantages for operational devices.^2^, ^3^, ^4^, ^5^ However, a few key challenges remain if perovskite solar cells are to move toward commercial manufacture, as for example long‐term stability, current–voltage hysteresis, and up‐scaling issues.^6^, ^7^, ^8^, ^9^, ^10^ Some excellent progress has been made on the issue of stability, with recent developments suggesting that perovskite solar cells may now deliver stable output for a few thousand hours under 1 sun (AM 1.5G) illumination.^11^, ^12^ Measures to improve stability include the use of mixed‐cation perovskites,^13^ and two‐dimensional (2D)^14^ and quasi‐2D perovskites^11^, ^12^ that incorporate spacer layers. Furthermore, efficient large‐area perovskite solar cells and modules have been successfully demonstrated,^15^, ^16^ indicating incipient success of up‐scaling approaches.

**Figure 1:**
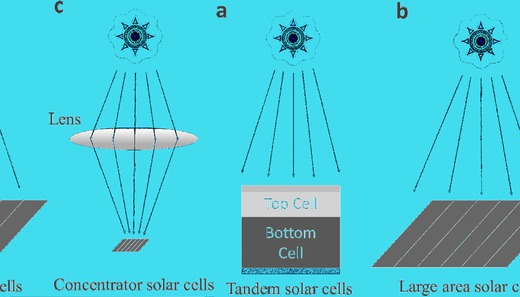
Schematic illustration of the working principles of a) tandem solar cells, b) large area solar cells, and c) concentrator solar cells.

As a result of such rapid improvements, perovskite solar cells are already approaching thermodynamically achievable efficiencies. These developments have recently triggered intense activities on measures designed to go beyond the Shockley–Queisser limit predicted for a single‐junction cell under standard solar illumination.^17^, ^18^ One strategy in this area is the fabrication of tandem solar cells,^13^, ^19^, ^20^, ^21^ whose dual‐absorber design allows the utilization of low‐energy photons without the open‐circuit voltage (*V*_oc_) losses otherwise incurred in a single‐junction cell. **Figure** **1**a schematically demonstrates the typical device architecture of a tandem solar cell, comprising a top cell of large‐bandgap absorber and a bottom cell based on a small‐bandgap material. However, fabrication of multijunction devices is complicated and costly, requiring delicate processing protocols. Solution‐processing of perovskite and transport layers for such multilayer subcells is even more challenging, requiring, e.g., the use of orthogonal solvents. In addition, any accidental presence of pin holes or diffusion of interfacial materials may spoil the whole device. Finally, any potential integration of perovskite photovoltaics with existing technology such as silicon solar cells within a genuine two‐terminal architecture, may also require loss‐free interfacing with light‐management structures that currently enhance performance of silicon cells.

A highly interesting alternative, that has however received remarkably little attention so far in the field of perovskite solar cells, is the use of concentrating photovoltaics.^22^ In this approach, solar cells are combined with concentrators to operate at higher light intensity than that encountered under typical solar (AM 1.5G) illumination. Concentrating photovoltaics is a facile and effective way of further enhancing the conversion of solar energy, illustrated by the fact that such (nonperovskite) assemblies currently hold PCE world records.^23^, ^24^, ^25^ One advantage is that, compared with tandem solar cells, there is no additional effort required in the fabrication of the actual solar cell device. Naturally, the concentrator technique is also compatible with tandem devices, and widely used with efficient multijunction tandem solar cells,^25^, ^26^ thereby combining the best of both approaches.

Another benefit of concentrator solar cell assemblies is that the solar cell device area can be much smaller for a given energy output, which saves on material and fabrication costs to some extent. The alternative approach of just increasing the device area (as shown in Figure 1b) would of course also allow the collection of more photons and higher power output. However, a concentrator solar cell (Figure 1c) requires much smaller active area, normally operating in conjunction with a focusing lens or a parabolic mirror. In addition, our literature survey presented in Table S1 of the Supporting Information shows that, for a collection of other (nonperovskite) technologies such as silicon, GaAs and CIGS,^24^, ^26^, ^27^ single‐ and multijunction solar cells exhibit enhanced efficiencies under solar concentrator scenarios. As we discuss further below, this phenomenon derives from the increased charge‐carrier concentration present in the material under higher solar illumination. Such enhanced device performance is the key benefit of concentrating photovoltaics and, as shown in this paper, should allow perovskite concentrator solar cells to move beyond the Shockley–Queisser limit even with the use of single‐junction cells.

Given the success of the concentrator approach for many inorganic photovoltaic technologies, it seems surprising that this concept has so far received little attention in the field of perovskite photovoltaics. One reason for this could be the enhanced requirements for the absorber materials when used in a concentrator concept, which includes sufficiently low charge‐carrier recombination rates and even higher demands on stability. The latter issue of thermal and photostability of hybrid organic–inorganic perovskites (HOIPs) is still under much discussion and development. However, promising recent studies have suggested that HOIPs exhibit an increasing photoluminescence quantum yield (PLQY) with increasing illumination of up to a few thousand suns, suggesting a stable enhancement of performance under solar concentrator conditions may be practically feasible.^28^

Motivated by these factors, we assess in this work the prospects for perovskite photovoltaics under solar concentration. Since typical charge‐carrier recombination and extraction parameters are now well‐known for HOIP materials, we are able to simulate expected device performance under the assumption that light‐induced material degradation is absent. Based on an analysis of charge‐carrier dynamics, we are able to predict parameters critical to device performance of perovskite concentrator solar cells (PCSCs), including short‐circuit current (*J*_sc_), open‐circuit voltage (*V*_oc_), fill factor (FF), and PCE as a function of solar illumination intensity. We show that the PCE of PCSCs should clearly improve with increasing illumination, and ought to eventually overtake the Shockley–Queisser limit at a few tens to hundreds of suns irradiance, depending on the density of charge traps present.

## 2 Results and Discussion

Our evaluations presented in this work are based on basic rate equations used to determine charge‐carrier concentrations inside a device under different operating conditions. A range of different charge‐recombination and ‐extraction rate parameters are used as input to the calculations that reflect what has so far experimentally been determined for these materials. We assume for the purpose of these calculations that the HOIPs are sufficiently stable under high illumination conditions, exhibiting negligible degradation. Given the current rapid progress in device and materials engineering, the stability of HOIPs and related devices may well be improved so that they can ultimately operate under the much harsher concentrator conditions for an extended period. Hot carriers effects, which could potentially improve matters at high charge carrier densities,^29^, ^30^ are not included in this work, which is based on the assumption that electron‐ and hole‐transporting layers are optimized to extract carriers at normal lattice temperature. In order to simplify our model, we also excluded series resistance and shunt resistance in this work. Ideally, the effect of parasitic resistances can be minimized by a decrease in device area and an enhancement in the junction quality. In addition, we assume that reflection losses are minimal, which can be achieved through suitable surface coatings. Under these conditions, the only unavoidable efficiency losses are caused by defect‐mediated charge‐carrier recombination and device performance can be calculated based on charge extraction and recombination rate parameters.

We begin by examining the short‐circuit scenario, where solar cells are working at steady state with a constant supply, i.e., under a given degree of illumination. Here, the charge‐carrier density *n* within the device is at equilibrium and may be determined by the solution to the following rate equation

(1) $$\frac{dn}{dt}\;\;=\;\;G\;\;-\;\;k_{1}n\;\;-\;\;k_{2}n^{2}\;\;-\;\;k_{3}n^{3}\;\;-\;\;c_{\mathrm{ext}}n\;=\;0$$

where *G* stands for the charge‐generation rate resulting from illumination, *k*_1_, *k*_2_, and *k*_3_ are the monomolecular, bimolecular, and Auger recombination rate constants, respectively, and *c*_ext_ denotes the charge extraction rate.

To evaluate the charge‐carrier generation rate *G* we consider that the best HOIPs‐based devices exhibit superb external quantum efficiency and almost 100% internal quantum efficiency (IQE) implying perfect charge generation.^31^ In the absence of reflection losses, we may therefore safely convert the solar irradiance power directly to a value for the charge‐carrier generation rate. For prototypical lead iodide perovskites films of thickness 300 nm, the spectral integral involving the 1 sun solar reference spectrum (AM 1.5G) and the perovskite absorption coefficient spectrum^32^ yields a value of *G* ≈ 5 × 10^21^ cm^−3^ s^−1^.

The second set of parameters to be assessed is the charge‐carrier recombination rate constants, which are associated with different recombination mechanisms. Table S2 of the Supporting Information summarizes values reported^33^, ^34^, ^35^, ^36^, ^37^ for *k*_1_, *k*_2_, and *k*_3_ from experiments and theoretical calculations. Monomolecular Shockley–Read–Hall recombination is associated with defects that act as recombination centers, resulting in a value of *k*_1_ that depends strongly on materials quality and processing protocol. Values are generally determined through a measurement of charge‐carrier lifetimes in the regime of low charge‐carrier density, which can typically range from nano‐ to microseconds.^32^ Figure S1 of the Supporting Information presents such sample transients for high‐quality HOIP films based on photoluminescence (PL) decays, which yield monomolecular charge‐carrier recombination rates *k*_1_ of ≈10^6^–10^7^ cm^−3^ s^−1^. We particularly highlight the long lifetimes (≈1 µs, i.e., *k*_1_ = 10^6^ s^−1^) obtained for mixed‐cation HOIPs involving formamidinium and cesium. Bimolecular charge‐carrier recombination, on the other hand, is related to band‐to‐band recombination of free electrons with holes, which is intrinsically impossible to avoid. Table S2 of the Supporting Information shows a range of experimentally determined values of *k*_2_ covering a wide range of (≈0.6–14) × 10^−10^ cm^3^ s^−1^. Such variations in *k*_2_ may be caused by changes in band‐structure between different materials,^38^ or differences in photon reabsorption resulting from changes in light outcoupling, scattering or film thickness.^39^ The importance of Auger recombination has also been theoretically assessed,^32^ based on known values of *k*_3_ near 10^−28^ cm^6^ s^−1^, and was found to be insignificant up to charge‐carrier densities around 10^18^–10^19^ cm^−3^. Experimental studies have yielded consistent results showing that PLQY deteriorated only at light intensity equivalent to over 10000 suns, for which Auger recombination started to dominate the recombination process.^28^ Here, we choose a typical value of *k*_3_ = 10^−28^ cm^6^ s^−1^ for all calculations presented in this study, but note that Auger effects do not appreciably influence the value of the calculated parameters over the solar illumination range shown.

Lastly, we account for the charge‐carrier extraction rate *c*_ext_, which is barely reported in the literature as it does not represent a fundamental property of any given materials and can hence easily change with device architecture, interlayers, carrier density and also built‐in field.^40^ In order to estimate a typical value for the charge extraction rate from HOIPs we recorded time‐resolved PL transients for a typical HOIP interfaced with several commonly used transport layers, such as [6,6]‐phenyl C61 butyric acid methyl ester (PC_61_BM), poly(3,4‐ethylenedioxythiophene):polystyrene sulfonate (PEDOT:PSS) or *N* ^2^,*N* ^2^,*N* ^2′^,*N* ^2′^,*N* ^7^,*N* ^7^,*N* ^7′^,*N* ^7′^‐octakis(4‐methoxyphenyl)‐9,9′‐spirobi[9H‐fluorene]‐2,2′,7,7′‐tetramine (Spiro‐MeOTAD). As shown in Figure S2 of the Supporting Information, the normally long‐lived carriers in HOIPs films can be effectively removed by both hole and electron transport layers resulting in PL quenching. As a basic approximation, we assume here a time‐independent charge‐carrier extraction rate, which may then be determined from such curves by the solution to the rate equation under very low light intensity

(2) $$\frac{dn}{dt}\;=\;-\;k_{1}n\;-\;c_{\mathrm{ext}}n$$

i.e., the PL decay curves are approximated as monoexponentials with a decay time corresponding to (*k*_1_ + *c*_ext_)^−1^. As shown in Figure S2 of the Supporting Information the shape of the quenched transients is not completely monoexponential, in particular at later time, which is expected, given that charge collection is limited by diffusion, which results in initially rapid charge extraction that slows down with time.^41^ However, since most charge‐carriers are collected with the equivalent extraction rate dominating the initial stage, we consider that an assumed extraction rate in the range of 10^8^–10^9^ s^−1^ reflects a sensible value in a constantly operated device. Furthermore, a high charge‐carrier concentration within the device could also result in higher built‐in fields that induce higher charge‐carrier extraction rates. We would hence consider a value of *c*_ext_ = 1 × 10^8^ s^−1^ as a reasonable approximation that should be justifiable in most cases and not unduly overestimate the device performance, but we will explore a wide range below.

**Figure 2:**
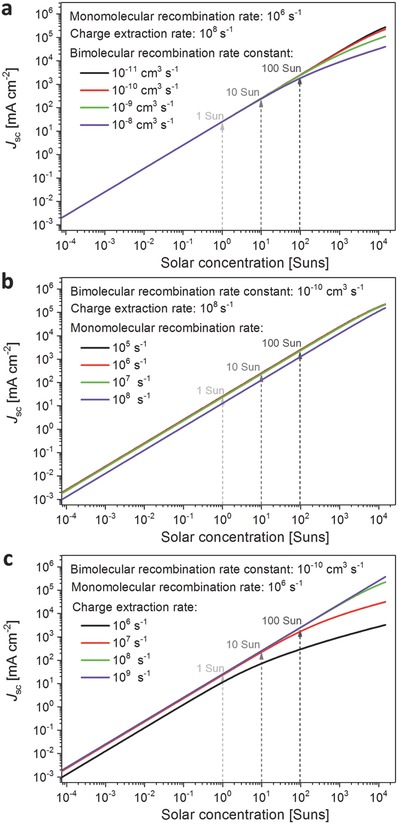
Short‐circuit current *J*_sc_ calculated as a function of solar concentration level for typical single‐junction perovskite solar cells (1.6 eV bandgap), for variation of a) the bimolecular charge‐carrier recombination rate constant *k*_2_, b) the monomolecular (trap‐mediated) recombination rate *k*_1_, and c) the charge extraction rate *c*_ext_. Unvaried parameters were set to *k*_1_ = 10^6^ s^−1^, *k*_2_ = 10^−10^ cm^3^ s^−1^, *k*_3_ = 10^−28^ cm^6^ s^−1^, and *c*_ext_ = 10^8^ s^−1^ for the calculations.

After our detailed discussion of such rate constants, we now turn to calculate *J*_sc_ from the extracted charge‐carrier density by solving Equation (1). The IQE (Equation ((3))) and *J*_sc_ (Equation ((4))) can then simply be calculated from the charge‐carrier extraction and generation rates, and the charge‐carrier density, as

(3) $$\mathrm{IQE}\;\;=\;\;\frac{c_{\mathrm{ext}}n}{G}$$

(4) $$J_{\mathrm{sc}}\;\;=\;\;qc_{\mathrm{ext}}\;nd$$

where *q* is elementary charge and *d* = 300 nm is the film thickness. **Figure** **2** displays the resulting values for *J*_sc_ as a function of solar illumination levels, with corresponding curves for the IQE provided in Figure S3 of the Supporting Information. These figures show in three panels the effect of variation in (a) bimolecular, (b) monomolecular, and (c) extraction rate constants, when the other two parameters are held at sensible reference values of *k*_1_ = 10^6^ s^−1^, *k*_2_ = 10^−10^ cm^3^ s^−1^, and *c*_ext_ = 10^8^ s^−1^.

Figure 2a shows that, not surprisingly, at low light intensity the *J*_sc_ depends linearly on the solar concentration. Deviations from linearity become apparent around 100 suns, where bimolecular charge‐carrier recombination begins to dominate, with higher values of *k*_2_ leading to both stronger deviations from linearity and its onset at lower solar concentration levels. By contrast, changes in monomolecular recombination rates (Figure 2b) do not have much of an effect on linearity but influence the responsivity, that is, the ratio between photoinduced current and illumination power. Figure 2c further demonstrates the competition between charge‐carrier extraction and recombination. With an increase in charge‐extraction rate, both bimolecular and monomolecular recombination losses are reduced, as also apparent in the recovery of the calculated IQE displayed in Figure S3c of the Supporting Information. We note that these findings are qualitatively consistent with recent light‐intensity dependent photocurrent measurements observed for organic solar cells.^42^, ^43^ Overall, the calculated relationship of the *J*_sc_ versus solar concentration level demonstrates that for typical charge‐carrier recombination and extraction parameters, optimized perovskite solar cells should have minimal deterioration of the *J*_sc_ up to 100 suns.

We next turn to the question of how the open‐circuit voltage *V*_oc_ is expected to develop with increasing solar concentration. Under open‐circuit conditions, there is no charge extraction from the device (*c*_ext_ = 0) and Equation (1) becomes

(5) $$G\;=\;k_{1}n\;\;+\;\;k_{2}n^{2}\;\;+\;\;k_{3}n^{3}$$

from which the carrier concentration *n* for any given charge‐carrier generation rate can be obtained. *V*_oc_ is highly dependent on *n* and can be expressed as

(6) $$qV_{\mathrm{oc}}\;\;=\;\;E_{g}\;\;-\;\;k_{B}T\;\ln\frac{N_{C}N_{V}}{n^{2}}$$

**Figure 3:**
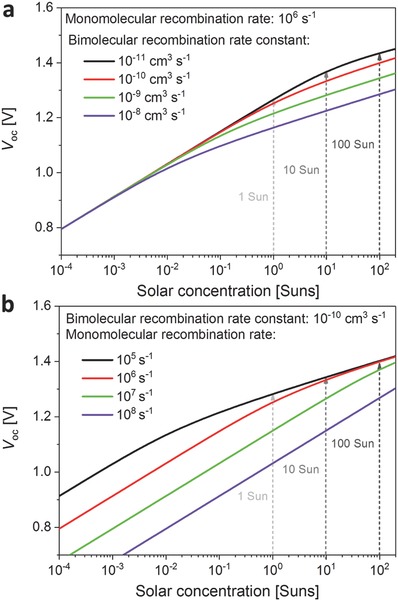
Open‐circuit voltage *V*_oc_ calculated as a function of solar concentration level for a typical single‐junction perovskite solar cells (1.6 eV bandgap), for variation of a) the bimolecular charge‐carrier recombination rate constant *k*_2_ at a fixed value of *k*_1_ = 10^6^ s^−1^, and b) the monomolecular (trap‐mediated) recombination rate *k*_1_ for fixed values of *k*_2_ = 10^−10^ cm^3^ s^−1^ and *k*_3_ = 10^−28^ cm^6^ s^−1^.

Here, *E*_g_ is the bandgap of the HOIP in question, for which we choose 1.6 eV as a representative value for materials incorporated into high‐efficiency perovskite solar cells, *k*_B_ is the Boltzmann constant, *T* is the temperature, and *N*_C_ and *N*_V_ are the effective number density of accessible states at the bottom of the conduction band and top of the valence band, respectively.^37^, ^44^ For the calculations below, we use estimated values of *N*_C_ = 2.7 × 10^18^ cm^−3^ and *N*_V_ = 3.9 × 10^18^ cm^−3^ based on assumed effective masses of electrons (m* ≈ 0.23*m*_0_) and holes (m* ≈ 0.29*m*_0_), respectively, as described previously,^37^, ^45^ using the relationship

(7) $$N_{C,V}\;\;=\;\;2{\left(\frac{2\pi m^{*}k_{B}T}{h^{2}}\right)}^{\frac{3}{2}}$$

where m* is the effective mass of electron or hole and *h* is the Planck constant. Through substituting the value *n* calculated from Equation (5) into Equation (6) we are able to determine the change of *V*_oc_ as a function of solar concentration, as shown in **Figure** **3**a,b.

Similar to the case of the short‐circuit voltage, we find that the *V*_oc_ increases with the light intensity. Figure 3a indicates that sensible changes in the values of the bimolecular recombination rate constant do not much affect *V*_oc_ under normal (unconcentrated) solar illumination conditions (<1 sun). Rather, deviations only occur under concentrated solar scenarios (>1 sun) for which bimolecular charge‐carrier recombination mechanisms begin to dominate over trap‐mediated recombination. Figure 3b shows that the low illumination regime is indeed dominated by the trap‐mediated charge‐carrier recombination rate *k*_1_. As the value of *k*_1_ is varied between 10^5^ s^−1^ (τ = *k*_1_^−1^ = 10 µs) and 10^8^ s^−1^ (τ = 10 ns) the *V*_oc_ changes by up to several hundred meV. For relatively low charge‐carrier lifetimes near τ = 10–100 ns, such Shockley–Read–Hall recombination losses persist well into the regime of solar concentration (10–100 suns). Figure S4a of the Supporting Information further illustrates this issue, displaying the *V*_oc_ versus the charge‐carrier lifetime τ = *k*_1_^−1^ at various solar concentrations. Figure S4b of the Supporting Information also displays the ideality factor derived from the slope of the *V*_oc_ versus solar‐concentration curves (as shown in Figure 3b). In the low‐illumination regime, an ideality factor near *m* = 2 indicates predominant Shockley–Read–Hall recombination, which gradually merges toward bimolecular recombination mechanisms (*m* = 1) as the illumination level moves toward the solar concentrator regime. These calculations reveal that higher irradiance will clearly improve the *V*_oc_, and that low trap‐related recombination rates are crucial for achieving high‐*V*_oc_ devices. For these reasons, optimized mixed‐cation perovskites normally allow for higher *V*_oc_ values, as these materials are currently more likely to exhibit particularly low nonradiative recombination losses.^37^, ^46^

**Figure 4:**
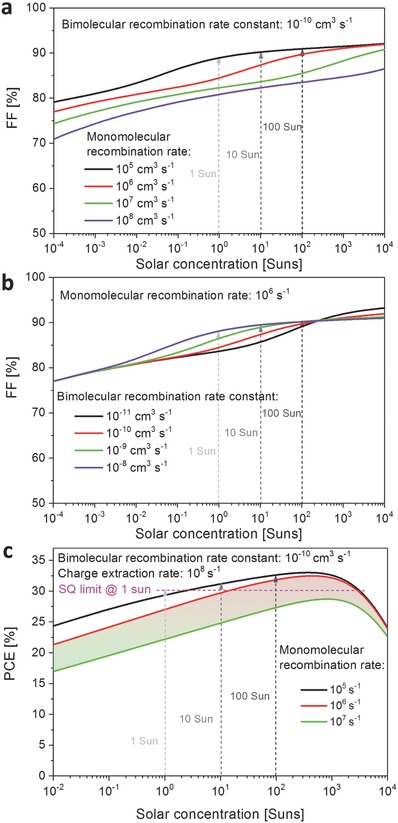
Fill factor (FF) as a function of solar concentration level for a typical single‐junction perovskite solar cell (1.6 eV bandgap), calculated based on Suns‐*V*_oc_ method, for variation of a) the bimolecular charge‐carrier recombination rate constant *k*_2_ at a fixed value of *k*_1_ = 10^6^ s^−1^, and b) the monomolecular (trap‐mediated) recombination rate *k*_1_ for a fixed value of *k*_2_ = 10^−10^ cm^3^ s^−1^. c) The calculated power‐conversion efficiency (PCE) for *k*_2_ = 10^−10^ cm^3^ s^−1^ and a charge‐extraction rate *c*_ext_ = 10^8^ s^−1^, for various monomolecular recombination rates *k*_1_. The Auger rate constant was set to *k*_3_ = 10^−28^ cm^6^ s^−1^ for all calculations. The Shockley–Queisser (SQ) limit^17^ of 30% for a single‐junction cell under 1 sun, based on a 1.6 eV bandgap absorber, is indicated by the pink dashed line.

Having established the dependence of *J*_sc_ and *V*_oc_ on the illumination intensity, we now turn to the most complex but vital parameter, i.e., the fill factor‐defining (FF) representing the “square‐ness” of the current–voltage characteristics. The FF is highly dependent on the competition between charge‐carrier recombination and extraction, which is influenced by device operation. We reiterate that the extraction rate is generally not a constant and highly dependent on the charge‐carrier density and the built‐in field, which brings uncertainty to the solution of the diode equation. The proximity of the FF to the recombination limit can be evaluated by a Suns‐*V*_oc_ method,^37^, ^44^ i.e., the pseudo‐*J*–*V* curves can be constructed from the light‐intensity dependent *V*_oc_ (Figure 3) by

(8) $$J\;\left(V_{\mathrm{oc}}\left(I\right)\right)\;\;=\;\;J_{\mathrm{sc}}\;\;\left(I_{0}\right)\;\;\cdot \;\;\left(1\;\;-\;\;\frac{I}{I_{0}}\right)$$

where *I* is the varied light intensity and *I*_0_ is the particular light intensity of the pseudo‐*J*–*V* curves. Here, parasitic resistance effects can be neglected since the *V*_oc_ is measured under open‐circuit condition. Typical calculated *J*–*V* curves for a various values of *k*_1_ and a value of *k*_2_ = 10^−10^ cm^3^ s^−1^ at 118 suns are shown in Figure S5 of the Supporting Information. Hence, the FF can be calculated based on the maximal power point (MPP) of these *J*–*V* curves as follows

(9) $$\mathrm{FF}\;\;=\;\;\frac{J_{\mathrm{mpp}}\;\;\cdot \;\;V_{\mathrm{mpp}}}{J_{\mathrm{sc}}\;\;\cdot \;\;V_{\mathrm{oc}}}$$

where *J*_mpp_ and *V*_mpp_ are the current density and voltage at maximal power point. Similarly, the FF at various light intensity and recombination rates can be assessed. **Figure** **4**a,b shows the resulting dependence of the FF on solar concentration level. It is evident that trap‐mediated monomolecular recombination is detrimental to the FF over the whole range of solar concentration of interest, while bimolecular recombination only affects the FF at higher intensities.

To ensure that we are able to capture the changes in FF with reasonable approximations, another empirical approach^47^ was utilized here to verify the obtained FF based on the expression between FF and *V*_oc_ provided by

(10) $$\mathrm{FF}\;\;=\;\;\frac{V_{\mathrm{oc},n}\;\;-\;\;\ln\left(V_{\mathrm{oc},n}\;\;+\;\;0.72\right)}{V_{\mathrm{oc},n}\;\;+\;\;1}$$

Here, *V*_oc,_ *_n_* is the open‐circuit voltage as normalized with respect to the thermal voltage *mk*_B_ *T*/*q*, where *m* is the ideality factor extracted from the dependence of *V*_oc_ on illumination intensity (see Figure S4b, Supporting Information). The results are presented in Figure S6 of the Supporting Information, and we observed almost identical trends.

We note that the trends obtained for the FF match well with those observed in *V*_oc_ (Figure 3) as a function of illumination. However, this would not necessarily be the case in reality because the series resistance will significantly hinder charge‐carrier extraction at higher concentrations and cause additional recombination, which will suppress the FF. Hence, any experimentally observed FF should exhibit a significant drop at the point where the current flow of the device encounters either the charge‐extraction limit associated with the sheet resistance of the transparent conductive electrodes (TCEs), or the space‐charge‐limited current of the interlayers and active layer. In addition, material degradation caused by thermal and/or photoinstability under high light intensity and temperature may also in reality suppress the FF.

Finally, we are able to calculate the PCE in the absence of optical losses based on the obtained values for *J*_sc_, *V*_oc_, and the FF with the usual equation

(11) $$\mathrm{PCE}\;\;=\;\;\mathrm{FF}\;\;\cdot \;\;J_{\mathrm{sc}}\;\;\cdot \;\;V_{\mathrm{oc}}/(1\;\mathrm{kW}\;m^{-2}\;\;\cdot \;\;\mathrm{Suns})$$

where Suns indicates the factor by which the incident full‐sun (AM 1.5) intensity is increased or reduced.

Figure 4c shows the expected PCE as a function of solar concentration (Suns) for a range of different monomolecular (trap‐mediated) recombination rates, based on typical values of *k*_2_ = 10^−10^ cm^3^ s^−1^ and *c*_ext_ = 10^8^ s^−1^. The PCE increases with the light intensity and peaks around a few hundred suns' concentration, beyond which it decreases as a result of increasing bimolecular recombination. The graph also displays the 30% maximum PCE limit calculated under the assumptions of Shockley and Queisser's theory for a material with bandgap of 1.6 eV under 1 sun AM 1.5G.^17^ Figure 4c demonstrates that for trap‐mediated recombination rates slightly below 10^7^ s^−1^ (or lifetimes in excess of a few hundred nanoseconds) the Shockley–Queisser limit can clearly be broken for a range of solar concentrations. In particular, for charge‐carrier lifetimes around 1 µs, which are already being achieved by many groups including our own (see Figure S1, Supporting Information), the Shockley–Queisser limit will be exceeded for the concentrator range of 10–100 suns. Furthermore, making such enhancements commence at illumination levels slightly above 1 Sun would only require a relatively modest increase in charge‐carrier lifetimes to 10 µs. Hence, provided the perovskite materials and related devices are stable enough, solar concentrators will offer a highly promising concept for perovskite solar cells operating beyond the Shockley–Queisser limit.

## 3 Conclusion

In summary, we have demonstrated that perovskite solar cells will fundamentally harvest photons more efficiently at certain regimes of high solar concentration, where they should be able to exceed the Shockley–Queisser limit and reach extraordinarily high open‐circuit voltages close to 1.4 V (i.e., losses limited to near 200 meV). Suppression of trap‐mediated (Shockley–Read–Hall) recombination of charge‐carriers is the key toward achieving this goal, but we argue that the required level is already approached, with charge‐carrier lifetimes in the low‐density regime now often exceeding microseconds. We therefore conclude that material and device stability under increased illumination levels would be the main challenge toward implementation of perovskite concentrator solar cells. Some key issues will need to be addressed in this regard, including the thermal and photostability of HOIPs and interlayers, and the long‐term stability of the devices. Moreover, there is clear room for improvement in the electrical sheet resistance of the TCEs, and the charge‐carrier mobility of the interlayers that are currently typically used. These issues could potentially be addressed by better control over doping and minimization of the effective thicknesses of such auxiliary layers. We note that such obstacles will in any case also have to be addressed for long‐term stable performance of perovskite solar cells under standard conditions. Most PV technologies that have achieved such stable operation in the field (e.g., silicon and GaAs) ultimately also qualified for solar concentrator applications. Therefore, we believe that further development of perovskite solar cells has the potential to ultimately allow their use in concentrating photovoltaics. Finally, the use of perovskite tandem cells under solar concentration will be a highly promising goal, given that the most efficient photovoltaic devices are currently working as a tandem structure under concentrator conditions.

## Conflict of Interest

The authors declare no conflict of interest.

## Supporting information

Supplementary
