# Supplementary material for: Hybrid Perovskites: Prospects for Concentrator Solar Cells
Source: Adv Sci (Weinh). 2018 Feb 1;5(4):1700792. doi: 10.1002/advs.201700792 (PMC5908368; doi:10.1002/advs.201700792)
Supplement: Supplementary file 1 — Supplementary [file ADVS-5-1700792-s001.pdf]

Copyright WILEY-VCH Verlag GmbH & Co. KGaA, 69469 Weinheim, Germany, 2018.

ADVANCED SCIENCE
Open Access

Supporting Information

for *Adv. Sci.,* DOI: 10.1002/advs.201700792

Hybrid Perovskites: Prospects for Concentrator Solar Cells

*Qianqian Lin, Zhiping Wang, Henry J. Snaith, Michael B. Johnston, and Laura M. Herz**

Copyright WILEY-VCH Verlag GmbH & Co. KGaA, 69469 Weinheim, Germany, 2016.

Supporting Information

Hybrid Perovskites: Prospects for Concentrator Solar Cells

Qianqian Lin[1,2], *Zhiping Wang*[1], *Henry J. Snaith*[1], *Michael B. Johnston*[1]

and Laura M. Herz[1]*

[1]Department of Physics, University of Oxford, Clarendon Laboratory, Parks Rd, Oxford, OX1 3PU, UK

[2]School of Physics and Technology, Wuhan University, Wuhan 430072, P. R. China

Corresponding Author：

Prof Laura M. Herz

*E-mail: laura.herz@physics.ox.ac.uk

Contents:

1. Experimental details

2. Supporting tables and figures

1. Experimental details

Preparation of perovskite films. $MAPbI_3$ solution: 1 M perovskite precursor solution was prepared using a 4 : 1 (v : v) mixed solvent from anhydrous dimethylformamide (DMF) and dimethyl sulfoxide (DMSO) from Sigma-Aldrich with desired composition using precursor salts: methylammonium iodide (MAI; Dyesol), lead iodide (PbI_2; TCI), lead bromide ($PbBr_2$; TCI). $FA_{0.83}Cs_{0.17}PbI_{2.7}Br_{0.3}$ solution: 0.6 M perovskite precursor solution was prepared using a 4 : 1 (v : v) mixed solvent from anhydrous DMF and DMSO (Sigma-Aldrich) with desired composition using precursor salts: formamidinium iodide (FAI; Dyesol), cesium iodide (CsI; Alfa Aesar), lead iodide (PbI_2; TCI), lead bromide ($PbBr_2$; TCI). $FA_{0.7}MA_{0.25}Cs_{0.05}PbI_{2.7}Br_{0.3}$ solution: 0.6 M perovskite precursor solution was prepared using a 4 : 1 (v : v) mixed solvent from anhydrous DMF and DMSO (Sigma-Aldrich) with desired composition using precursor salts: formamidinium iodide (FAI; Dyesol), methylammonium iodide (MAI; Dyesol), cesium iodide (CsI; Alfa Aesar), lead iodide (PbI_2; TCI), lead bromide ($PbBr_2$; TCI). All the solutions were made in a nitrogen-filled glovebox and kept stirring overnight at room temperature. The $MAPbI_3$ films were deposited through a two-step spin coating program (10 s at 1000 rpm and 15 s at 6000 rpm) with dripping of anisole (Sigma-Aldrich) as anti-solvent during the second step, 10 s before the end. The films were then transferred to a hotplate immediately afterwards and annealed at 100 °C for 10 min. For films containing the FA-Cs and FA-MA-Cs mixtures, the precursor solutions were spin-coated through a two-step spin coating program (10 s at 1000 rpm and 35 s at 6000 rpm) with dripping of anisole (Sigma-Aldrich) as anti-solvent during the second step, 10 s before the end. The films were then annealed at 100 °C for 60 min. All the films were spin-coated in a drybox with relative humidity below 20%.

Preparation of quencher layers on perovskite films. [6,6]-phenyl C61 butyric acid methyl ester (PC61BM, Solenne) was dissolved in Chloroform at 30 mg mL^{-1} and spin-coated at

1000 rpm for 30 s. Poly(3,4-ethylenedioxythiophene):polystyrene sulfonate (PEDOT:PSS, Heraeus) was spin-coated at 2000 rpm for 60s, and $N^2,N^2,N^{2'},N^{2'},N^7,N^7,N^{7'},N^{7'}$-octakis(4-methoxyphenyl)-9,9'-spirobi[9H-fluorene]-2,2',7,7'-tetramine (Spiro-MeOTAD) solution was spin-coated at 2500 rpm for 40s. To obtain a spiro-OMeTAD solution, we dissolved 85.7mg spiro-OMeTAD (Borun Technology) in 1ml anhydrous chlorobenzene with additives of 28.8 μl tert-butylpyridine (tBP) and 20 μl lithium bis(trifluoromethylsulfonyl) imide (Li-TFSI) salt in acetonitrile (520 mg ml^{-1}).

Characterization of PL lifetime. PL spectra were acquired using a time-resolved single photon-counting set-up (FluoTime 300, PicoQuant). Samples were photo-excited using a 507 nm laser head (LDH-P-C-510, PicoQuant) with pulse duration of 117 ps, fluence of ~30 nJ cm^{-2} per pulse and a repetition rate of 1 MHz. The PL was collected using a monochromator and hybrid photomultiplier detector assembly (PMA Hybrid 40, PicoQuant GmbH).

2. Supporting tables and figures

Table S1. Comparison of the record efficiencies of standard (one-sun) and concentrator solar cells.

Classification	One-sun solar cell	Concentrator solar cell	
	Efficiency [%]	Efficiency [%]	Solar Concentration [Suns]
Silicon	25.63[a]	27.6[b]	92
GaAs	28.8 [b]	29.1[b]	117
CIGS	22.6[a]	23.3[b]	15
Multi-junction cells	38.8 [c]	46.2[b]	508

a data taken from Ref. S1; **b** data taken from Ref. S2; **c** data taken from Ref. S3.

Table S2. Typical recombination rate parameters for hybrid organic-inorganic metal halide perovskites.

Perovskite formula	Monomolecular recombination rate [s^{-1}]	Bimolecular recombination rate constant [$cm^3\ s^{-1}$]	Auger recombination rate constant [$cm^6\ s^{-1}$]	
$CH_3NH_3PbI_3$	15×10^{6}	0.6×10^{-10}	1.6×10^{-28}	Ref. S4
$CH_3NH_3PbI_{3-x}Cl_x$	5×10^{6}	0.9×10^{-10}	1×10^{-28}	Ref. S5
$CH(NH)_2PbI_3$	7×10^{6}	1.1×10^{-10}	0.2×10^{-28}	Ref. S6
$CH_3NH_3SnI_3$	8×10^{9}	14×10^{-10}		Ref. S7
$CH_3NH_3PbI_3$ (calculated)		$0.1\sim1 \times 10^{10}$		Ref. S8
$CH_3NH_3PbI_3(Cl)$	$\sim1 \times 10^{6}$			Ref. S9

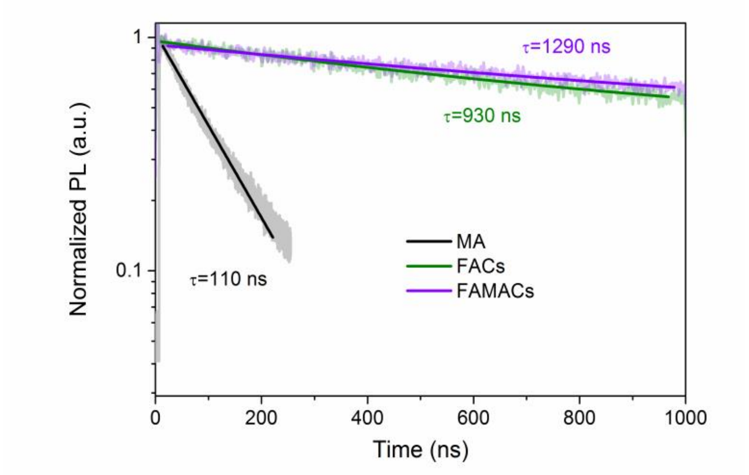

Figure S1. Photoluminescence transients for thin films of various hybrid organic-inorganic perovskites, including a methylammonium (MA) perovskite ($MAPbI_3$), a formamidinium cesium (FACs) double-cation perovskite ($FA_{0.83}Cs_{0.17}PbI_{2.7}Br_{0.3}$) and a formamidinium – methylammonium-cesium (FAMACs) triple cation perovskite ($FA_{0.7}MA_{0.25}Cs_{0.05}PbI_{2.7}Br_{0.3}$). Data were taken using the TCSPC technique, following low-fluence pulsed excitation at a wavelength of 507nm. The lifetime values indicated for τ are derived from mono-exponential fits to the transients.

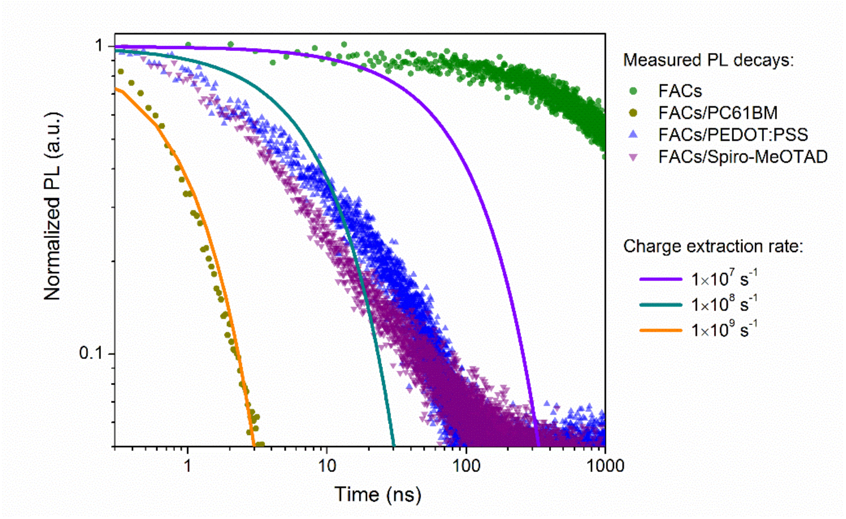

Figure S2. PL quenching transients measured for a thin film of the formamidinium cesium (FACs) double cation perovskite ($FA_{0.83}Cs_{0.17}PbI_{2.7}Br_{0.3}$) covered with a layer of various

transport materials, shown together with calculated curves based on various constant (time-independent) charge extraction rates (solid lines).

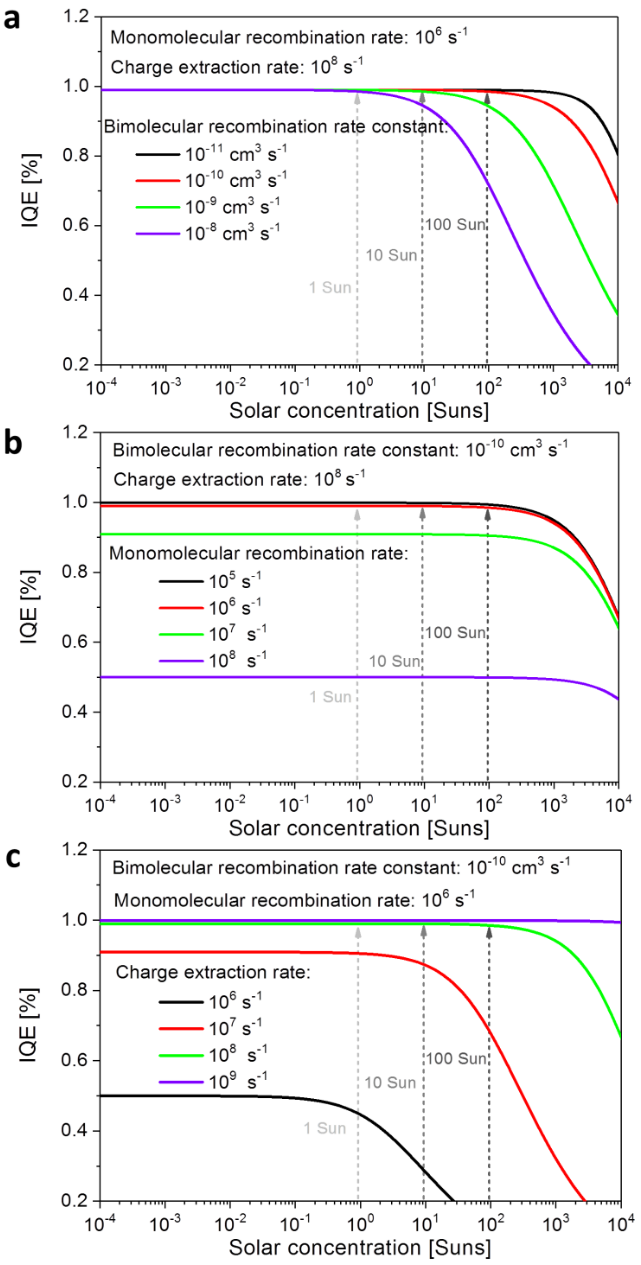

Figure S3. Internal quantum efficiency (IQE) calculated as a function of solar concentration level for a typical single-junction perovskite solar cells (1.6eV bandgap), for variation of (**a**) the bimolecular charge-carrier recombination rate constant k_2, (**b**) the monomolecular (trap-mediated) recombination rate k_1 and (**c**) the charge extraction rate c_{ext}. Unvaried parameters were set to $k_1=10^6$ s^{-1}, $k_2=10^{-10}$ cm^3s^{-1}, $k_3=10^{-28}$ cm^6s^{-1} and $c_{ext}=10^8$ s^{-1} for the calculations.

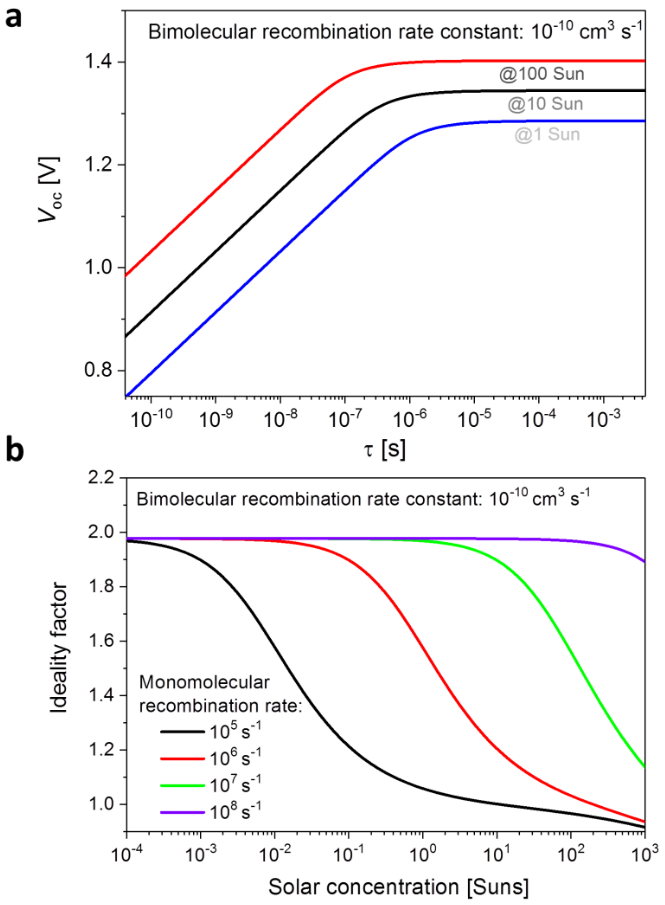

Figure S4. (**a**) Open circuit voltage as a function of charge-carrier lifetime $\tau=k_1^{-1}$ for a typical single-junction perovskite solar cell, calculated for a number of different solar concentration levels. (**b**) Ideality factor *m*, for a range of different monomolecular recombination rates k_1, as derived from the slope of the curves shown in **Figure 3b** that relate the open-circuit voltage V_{oc} to the Solar concentration (Suns), where $m= (q/k_BT)*d(V_{oc})/d(\ln(Suns))$,[S10] and *Suns* is the illumination intensity normalized to that under standard solar (AM1.5) conditions. The Auger rate constant was set to $k_3=10^{-28}$ cm^{6}s^{-1} for all calculations.

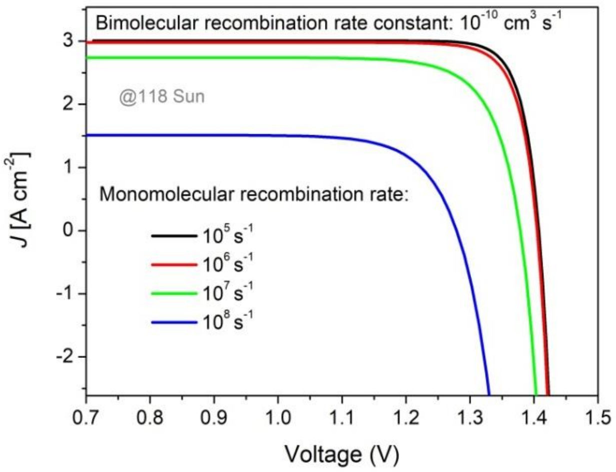

Figure S5. Calculated J-V curves based on the Suns-V_{oc} method at an illumination of 118 Sun, for variation of the monomolecular recombination rate k_1 for fixed values of k_2=10^{-10} cm^{3}s^{-1} and k_3=10^{-28} cm^{6}s^{-1}.

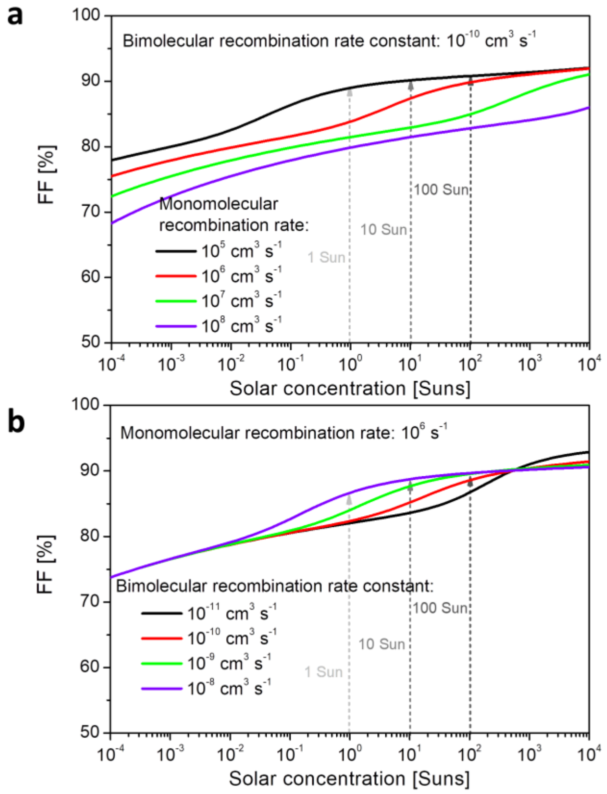

Figure S6. Fill factor as a function of solar concentration level calculated based on empirical relationship between FF and V_{oc},[S11] for variation of (**a**) the bimolecular charge-carrier recombination rate constant k_2 at a fixed value of k_1=10^6 s^{-1}, and (**b**) the monomolecular (trap-

mediated) recombination rate k_1 for a fixed value of k_2=10^{-10} cm^3s^{-1}. The Auger rate constant was set to k_3=10^{-28} cm^6s^{-1} for all calculations.

References:

[S1]. Green, M. A.; Emery, K.; Hishikawa, Y.; Warta, W.; Dunlop, E. D., *Prog. Photovoltaics,* **2015**, *23*, 1-9.

[S2]. NREL Efficiency Chart, https://www.nrel.gov/pv/assets/images/efficiency-chart.png, accessed on 15th, July 2017

[S3]. P. Chiu, D. Law, R. Woo, S. Singer, D. Bhusari, W. Hong, A. Zakaria, J. Boisvert, S. Mesropian, R. King, "35.8% Space and 38.8% Terrestrial 5J Direct Bonded Cells", presented at *Photovoltaic Specialist Conference (PVSC), 2014 IEEE 40th*, 2014.

[S4]. Milot, R. L.; Eperon, G. E.; Snaith, H. J.; Johnston, M. B.; Herz, L. M., *Adv. Funct. Mater.,* 2015, *25*, 6218-6227.

[S5]. Wehrenfennig, C.; Eperon, G. E.; Johnston, M. B.; Snaith, H. J.; Herz, L. M., *Adv. Mater.,* **2014**, *26*, 1584-1589.

[S6]. Rehman, W.; Milot, R. L.; Eperon, G. E.; Wehrenfennig, C.; Boland, J. L.; Snaith, H. J.; Johnston, M. B.; Herz, L. M., *Adv. Mater.,* **2015**, *27*, 7938-7944.

[S7]. Noel, N. K.; Stranks, S. D.; Abate, A.; Wehrenfennig, C.; Guarnera, S.; Haghighirad, A.-A.; Sadhanala, A.; Eperon, G. E.; Pathak, S. K.; Johnston, M. B., *Energy Environ. Sci.,* **2014**, *7*, 3061-3068.

[S8]. Tress, W., Maximum Efficiency and Open-Circuit Voltage of Perovskite Solar Cells. In Organic-Inorganic Halide Perovskite Photovoltaics, Springer: 2016; p53-77.

[S9]. Vorpahl, S. M.; Stranks, S. D.; Nagaoka, H.; Eperon, G. E.; Ziffer, M. E.; Snaith, H. J.; Ginger, D. S., *Science,* **2015**, *348*, 683-686.

[S10]. Beattie N S, Zoppi G, See P, et al., *Sol. Energy Mater. Sol. Cells,* **2014**, *130*, 241-245.

[S11]. M. A. Green, Solid-State Electron. 1981, 24, 788.
